# Impact of Age, Multimorbidity and Frailty on the Prescription of Preventive Antiplatelet Therapy in Older Population

**DOI:** 10.3390/ijerph17124541

**Published:** 2020-06-24

**Authors:** Caroline Laborde, Jérémy Barben, Anca-Maria Mihai, Valentine Nuss, Jérémie Vovelle, Philippe d’Athis, Pierre Jouanny, Alain Putot, Patrick Manckoundia

**Affiliations:** 1 Department of Geriatrics and Internal Medicine, Hospital of Champmaillot, University Hospital, 21000 Dijon, France; caroline.laborde@chu-dijon.fr (C.L.); jeremy.barben@chu-dijon.fr (J.B.); anca.mi17@yahoo.com (A.-M.M.); valentine.nuss@chu-dijon.fr (V.N.); jeremie.vovelle@chu-dijon.fr (J.V.); pierre.jouanny@chu-dijon.fr (P.J.); patrick.manckoundia@chu-dijon.fr (P.M.); 2 Department of Biostatistics and Medical Information, François Mitterrand Hospital, University Hospital, 21000 Dijon, France; philippe.athis@chu-dijon.fr; 3 UMR Inserm/U1093 Cognition, Action, Sensorimotor Plasticity, University of Burgundy and Franche Comté, 21000 Dijon, France

**Keywords:** age, multimorbidity, frailty, overuse, underuse, antiplatelet agents, platelet aggregation inhibitors, elderly, comprehensive geriatric assessment

## Abstract

Platelet aggregation inhibitors (PAI) have widely proven their efficiency for the prevention of ischemic cardiovascular events. We aimed to describe PAI prescription in an elderly multimorbid population and to determine the factors that influence their prescription, including the impact of age, comorbidities and frailty, evaluated through a comprehensive geriatric assessment. This cross-sectional study included all patients admitted to the acute geriatric department of a university hospital from November 2016 to January 2017. We included 304 consecutive hospitalized patients aged 88.7 ± 5.5 years. One third of the population was treated with PAI. A total of 133 (43.8%) patients had a history of cardiovascular disease, 77 of whom were on PAI. For 16 patients, no indication was identified. The prescription or the absence of PAI were consistent with medical history in 61.8% of patients. In the multivariate analysis, among the 187 patients with an indication for PAI, neither age (odds ratio (OR) = 1.00; 95% confidence interval (CI): [0.91–1.08], per year of age), nor comorbidities (OR = 0.97; 95% CI: [0.75–1.26], per point of Charlson comorbidity index), nor cognitive disorders (OR = 0.98; 95% CI [0.91–1.06] per point of Mini Mental State Examination), nor malnutrition (OR = 1.07; 95% CI [0.96–1.18], per g/L of albumin) were significantly associated with the therapeutic decision. PAI were less prescribed in primary prevention situations, in patients taking anticoagulants and in patients with a history of bleeding. In conclusion, a third of our older comorbid population of inpatients was taking PAI. PAI prescription was consistent with medical history for 61.8% of patients. Age, multimorbidity and frailty do not appear to have a significant influence on therapeutic decision-making. Further research is needed to confirm such a persistence of cardiovascular preventive strategies in frail older patients from other settings and to assess whether these strategies are associated with a clinical benefit in this specific population.

## 1. Introduction

Cardiovascular diseases account for 45% of deaths in Europe, which amounts to more than 4 million deaths per year, 65% of which occur after the age of 65 [1]. Age is the most significant non-modifiable cardiovascular risk factor, and it is also a risk factor for recurrence [2]. In addition, cardiovascular events in the elderly result in a higher rate of long-term disability and dependence [3].

Platelet aggregation inhibitors (PAI) have been shown to be effective for the secondary prevention of cardiovascular disease, and this effect is maintained in older adults [4]. The benefit–risk ratio is less clearly established in primary prevention. In 2018, the ASPirin in Reducing Events in the Elderly (ASPREE) study demonstrated that the use of a PAI for primary prevention does not reduce the risk of cardiovascular events in the elderly, but it does increase the risk of major bleeding [5]. A meta-analysis published in 2019 found that the use of PAI in primary prevention was associated with a lower risk of cardiovascular events but a higher risk of major bleeding [6].

Prescribing drugs is particularly complex in the elderly, and vigilance is required with regard to both excess (overuse) and insufficient treatment (underuse) [7]. PAI is one of the most commonly underused drugs for secondary prevention in geriatrics [8], despite strong evidence that its use is beneficial [9]. However, there is to date little evidence of benefit of preventive strategies in frail older subjects with multiple comorbidities and polypharmacy. Several studies have shown a lower compliance with the recommendations for PAI when physicians treat older patients or patients with dependence or cognitive disorders [10,11,12]. Evidence shows an inverse relationship between cardiovascular preventive drugs prescription and age [13]. However, there is growing awareness of the importance of multimorbidity, and poor functional and cognitive status, rather than age itself, as factors associated with prognosis and underprescription in the elderly [14,15,16,17,18].

In light of these findings, we conducted a study to analyze the prescription of PAI among patients hospitalized in acute geriatrics. We assessed whether age, multimorbidity and frailty, evaluated by a comprehensive geriatric assessment (CGA), were independently associated with fewer PAI prescriptions.

## 2. Methods

### 2.1. Design

This monocentric observational cross-sectional study was conducted in the acute geriatric department of a university hospital over a 2-month period. The study was conducted in accordance with the Declaration of Helsinki and national standards, and with the approval of the ethics committee of our institution. Data were collected from the medical record, including the systematic CGA performed by the medical team.

### 2.2. Population

We included all consecutive patients admitted to the acute geriatric department from home, the emergency department or another acute care unit at the university hospital between 2 November 2016 and 6 January 2017. Hospitalization in the acute geriatric department during the study period was the unique inclusion criterion. We excluded readmissions over the study period and patients for whom data collected were unavailable in the medical record. There were no other exclusion criteria in the present study.

### 2.3. Data Collected

For each subject, we collected: age, sex, place of residence and the main medical history, including cardiovascular risk factors such as high blood pressure (hypertension), dyslipidemia, chronic kidney disease, diabetes and active smoking. We also collected chronic treatment at admission, including PAI and anticoagulants, history of severe bleeding (intracranial or digestive hemorrhage, hematuria or deep bleeding), transfusion of red blood cells within the previous 6 months, known iron deficiency, and hemoglobin level on admission. Polypharmacy was defined as the routine use of more than five medications [19]. In addition, multimorbidity, cardiovascular risk and CGA were performed for each patient. Consistency of PAI prescription with current guidelines was also established, according to the current French guidelines [20], in primary or secondary prevention, in case of high cardiovascular risk or symptomatic atherosclerosis (as defined in Section 2.3.3), respectively.

#### 2.3.1. Multimorbidity Assessment

Comorbidities were evaluated by the (non-age-adjusted) Charlson comorbidity index [21]. Multimorbidity was defined by the presence of two chronic diseases or more at admission [22].

#### 2.3.2. Comprehensive Geriatric Assessment

Items of the CGA were also collected [23]. The Mini-Mental State Examination (MMSE) score was used to divide subjects into 3 groups according to their score (≥21, between 10 and 20 or <10). A history of at least two falls in the previous year was documented. Gait speed, Tinetti test or minimum motor test were used to evaluate mobility. Motor fragility was defined as either a gait speed ≤ 0.70 m/s, a Tinetti test score ≤ 23/28 or a minimum motor test score < 15/20 [24]. Nutrition status was evaluated with the serum albumin level and according to the recommendations for the identification of adult malnutrition [25].

#### 2.3.3. Cardiovascular Risk Assessment

A patient was considered to be at high cardiovascular risk if he has complicated diabetes, a glomerular filtration rate < 45 mL/min or at least 3 of the following cardiovascular risk factors: age > 50 years in males and >60 years in females, hypertension, diabetes, active smoking and high-density lipoprotein (HDL) cholesterol ≤ 0.40 g/L [21]. Cardiovascular events defining symptomatic atherosclerosis were determined according to the French National Health Authority guidelines regarding the prescription of PAI in 2012 [20]: ischemic heart disease, non-cardioembolic stroke or transient ischemic attack, peripheral artery disease (PAD), other ischemic events or an endovascular procedure.

### 2.4. Statistical Analyses

Patients with and without a prescription for PAI were compared in univariate and multivariate analyses. The dichotomous variables were expressed in absolute numbers and percentages, while the quantitative variables were expressed as means and standard deviations in case of normal distribution or as medians and interquartile ranges otherwise. In the univariate analysis, the chi-square test was used to compare qualitative values. Quantitative values were analyzed using the Student’s t-test. Multivariate analysis with logistic regression was also performed in cases of *p* < 0.1 in the bivariate analysis or clinical significance. Significance was set at *p* < 0.05. SPSS version 23 (IBM Inc., Armonk, NY, USA) was used for all statistical analyses.

## 3. Results

### 3.1. Population

Three-hundred-and-thirty-three patients were admitted over the target period; 13 (3.9%) were excluded because their prescription data were missing and 16 (4.8%) because they were readmitted over the study period. Ultimately, 304 patients with a mean age of 88.7 ± 5.5 years (extremes 69 and 108 years) were included, of whom 174 (57.2%) were women. A total of 53 patients (17.4%) were nursing home residents. A majority of patients were considered multimorbid (non-age-adjusted Charlson score ≥ 2 for 188 patients (61.8%)). The following parameters of the CGA were highlighted:

- Cognitive evaluation: 267 patients were divided into three subgroups according to MMSE score: 100 patients (37.5%) had a score of ≥21/30, 122 (45.7%) a score between 10 and 20/30 and 45 (16.9%) a score < 10/20;
- Motor skills: In the previous year, 105 patients (34.5%) had experienced at least two falls. Of the 250 patients for whom the motor evaluation was performed, 211 (69.4%) had motor frailty. The mean gait speed was 0.37 ± 0.3 m/s. The mean minimum motor test score was 13.6 ± 3.9/20 and the mean Tinetti test score was 20 ± 5.1/28;
- Nutritional evaluation: The mean serum albumin was 29.2 ± 4.8 g/L, and 143 patients (48.8%) were found to have severe protein-energy malnutrition.

### 3.2. PAI Indications

Age was a cardiovascular risk factor for all patients. In addition, 217 (71.4%) had hypertension, 81 (26.6%) had dyslipidemia, 85 (28.0%) had diabetes and 5 (1.6%) were active smokers. Fifty-four patients (17.8%) had never had a cardiovascular event but had a high cardiovascular risk and could therefore have been treated with PAI for primary prevention.

One-hundred-and-thirty-three patients (43.8%) had a history of symptomatic atherosclerosis warranting PAI for secondary prevention. The indication was ischemic heart disease in 69 cases, non-cardioembolic stroke in 67 cases and symptomatic PAD in 18 cases. The other 21 indications were an endovascular procedure for 14 patients and another ischemic event in 7 cases (ischemic colitis, mesenteric ischemia or ischemic optic neuropathy). A total of 31 patients had already experienced several cardiovascular events.

Concerning PAI contraindications and risks, history of severe bleeding was recorded in 40 patients (13.2%). It was a digestive hemorrhage in 19 cases, an intracranial hemorrhage in 12 cases and another type (hematuria or deep bleeding) in 9 cases. These events had occurred in the previous year in 19 patients. One-hundred-and-thirty-one patients (43.1%) were anemic, 30 (9.9%) patients had hemoglobin levels ≤ 10 g/L, 9 (3%) had hemoglobin levels ≤ 8 g/dL and 17 had received a red blood cell transfusion in the six months prior to hospitalization.

### 3.3. PAI Prescription

On admission, 103 patients (33.9%) were taking PAI: 83 on aspirin alone (80.6%), 16 on clopidogrel alone (15.5%) and 4 (3.9%) on combined therapy (aspirin and clopidogrel in 3 cases and aspirin and ticagrelor in 1 case). Of these 103 patients, 77 were on PAI for secondary prevention, 10 for primary prevention and 16 had no indication (overuse). The 77 cases of symptomatic atherosclerosis (one or more associated cases) included 41 cases of coronary heart disease, 36 strokes, 15 PAD and 7 other indications.

**Figure 1:**
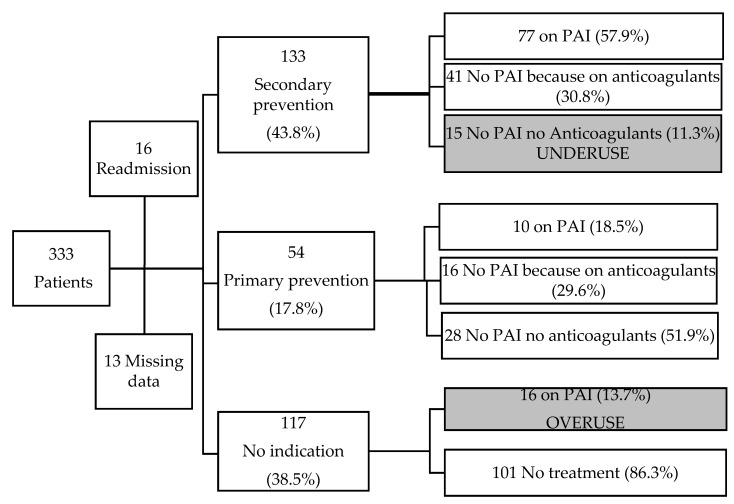
Flow chart. PAI: platelet aggregation inhibitors. The black text boxes indicate inconsistency with current guidelines [20].

The prescription or non-prescription of PAI was consistent with the patient’s medical history in 61.8% of the population (i.e., 188 patients: PAI for primary prevention for 10 patients, for secondary prevention for 77 patients, no indication of PAI for 101 patients). Figure 1 summarizes the treatments according to the patients’ level of cardiovascular risk.

In the univariate analysis of the 187 patients with an indication for PAI (Table 1), age, gender and nursing home resident status were not significantly associated with PAI prescription. The Charlson scores and rates of motor disorders were similar in the two groups. Patients classified as primary prevention or taking anticoagulants were less frequently on PAI, whereas patients with coronary artery disease were more frequently prescribed PAI. Proton pump inhibitors and statins co-prescriptions were significantly more frequent among patients on PAI.

In the multivariate analysis, age, multimorbidity and altered motor, cognitive or nutritional status did not significantly influence the prescription of PAI (Table 2). Polypharmacy was significantly more frequent in patients prescribed PAI (OR = 4.14; 95% CI [1.12–15.29]; *p* = 0.033). Patients with an indication for PAI as primary prevention were less likely to be treated (OR = 0.04; 95% CI [0.01–0.20]; *p* < 0.001). On the contrary, symptomatic PAD was more frequent in patients on PAI (OR = 5.30; 95% CI [1.07–26.2]; *p* = 0.041). Compliance with the recommendations regarding PAI was lower in patients with a history of bleeding and/or anticoagulant therapy (OR = 0.14; 95% CI [0.03–0.63]; *p* = 0.030 and OR = 0.01; 95% CI [0.004–0.05]; *p* < 0.001, respectively).

## 4. Discussion

The interest of this study lies in its exhaustive nature as well at the target population—an elderly multimorbid population taking multiple medications.

This work highlights the high prevalence of cardiovascular disease in the elderly considering that nearly a half of the population had an indication for PAI. Such epidemiological data are rare because most studies focus on specific indications for PAI and few consider the hospitalized elderly population as a whole. Here we provide a comprehensive record of the geriatric context associated with prescription or non-prescription of PAI.

### 4.1. PAI in Older Patients: from Underuse to Overuse

Treatments for cardiovascular disease are the most widely consumed drug class in the elderly population. This study shows that more than one third of a hospitalized geriatric population was on PAI, which is consistent with retrospective data obtained in an elderly French ambulatory population [26]. PAI prescription was consistent with medical history in 61.8% of patients. Despite strong supporting evidence, levels of compliance to the recommendations are often disappointing [27]. This is especially true in the geriatric population, where there is a paradoxical underuse of PAI in patients with a high risk of cardiovascular events [11,28,29]. In our work, the prescription of PAI was observed in only 58% of patients with an indication for PAI as secondary prevention (underuse). Long-term PAI have proven their effectiveness in symptomatic atherosclerosis, reducing cardiovascular mortality and severe cardiovascular morbidity by 25% [9]. This benefit is recognized as greater than the risk of bleeding [30]. Underuse of PAI for documented atherosclerosis is one of the most common instances of underuse in geriatrics [8]. One study showed that 40% of patients received neither PAI nor anticoagulants after stroke and a hospital stay in geriatrics [10]. Another investigation in patients discharging from hospital after myocardial infarction reported a PAI prescription in 88% of patients aged <75 years but only in 66% after the age of 84 [11]. Another study found that only a half of patients with a history of stroke or coronary artery disease had received a prescription for PAI prior to hospitalization [31].

Ten patients with a high cardiovascular risk were prescribed PAI for primary prevention, as recommended in the French guidelines from 2012 [20]. However, the Australian ASPREE randomized controlled trial, which compared the prescription of low-dose aspirin to a placebo in primary prevention, showed no benefit for cardiovascular mortality, disability or all cause of mortality in the PAI group [5]. Based on these data, the European Society of Cardiology (ESC) chose in 2016 to no longer recommend the use of PAI for primary prevention [32].

In our population, 16 of the 117 patients without identified cardiovascular risk factors were taking PAI without any indication (overuse). This is a frequent occurrence in geriatrics. A French study from 2011 found that, among 219 patients aged 70 years and older, 16.9% of PAI prescriptions were off-label [33]. Considering that 5% of patients have experienced an episode of bleeding, the authors concluded that it was unacceptable to put patients at such a risk.

### 4.2. Factors Associated with PAI Prescriptions

In our study, we did not find that patient characteristics, regardless of the medical history, led to changes in therapy. Notably, though patient age has previously been described as a risk factor for the underuse of PAI, it did not influence prescriptions in our population [27,31]. Underuse of beneficial medications is frequently reported for the oldest old, including for PAI [34]. In contrast, Filippi et al. found that prescriptions after stroke were more appropriate in patients aged > 65 years and in men [35]. Concerning the indications of PAI in secondary prevention, symptomatic PAD was associated with a higher rate of PAI prescription than other indications, including history of stroke and coronary artery disease. To our knowledge, there are no data in the literature to compare with these results. We may assume that pain related to symptomatic PAD could reinforce guidelines adherence, compared with asymptomatic pathologies.

Multimorbidity, evaluated by the Charlson index [21], was not associated with PAI underuse in our series. Our findings are contrary to a previous study that found a low Charlson index to be an independent predictive factor for the use of PAI in the acute phase of myocardial infarction [36]. More generally, multimorbidity has been associated with underuse of indicated medications in hospitalized American veterans [37]. However, the existence of systematic associations in drug prescription leading to the establishment of patterns of polypharmacy could dampen the underuse of PAI in multimorbid patients [38]. Interestingly, we found PAI prescription to be independently associated with polypharmacy. The frequent co-prescription of statins with PAI, highlighted in our series, has been already identified as belonging to a pattern of polypharmacy [38]. This association is clinically relevant as these two therapeutic classes have the same main indications (i.e., primary or secondary atherosclerosis prevention). Prevention of PAI-induced hemorrhagic risk by proton pump inhibitors in older patients, although not systematically recommended, is also common in practice [39] and increases the risk of polypharmacy in patients on PAI.

The CGA did not individualize any factor associated with PAI prescription. To our knowledge, such findings have never been published for PAI, but similar results were found for anticoagulants in a geriatric setting [40]. We found no link between motor disability or falls and the prescription of PAI. However, one study showed that patients taking PAI were at an increased risk of bleeding in the event of a fall [35]. In our work, cognitive disorders were not a barrier to the prescription of PAI, while an Italian study showed that patients with memory problems were less likely to be treated with PAI after hospitalization for stroke [10]. It appears that physician knowledge and experience, as well as their own perception of ageing, could influence therapeutic decision-making in older multimorbid patients [41,42,43]

In our study, there was a strong disinclination to prescribe both PAI and anticoagulants: in 30% of cases, the absence of PAI treatment when indicated was justified by the concomitant prescription of anticoagulants. In 2014, the ESC approved the discontinuation of PAI in patients with stable coronary artery disease treated with long-term anticoagulant therapy [10]. This attitude is in line with the data in the literature demonstrating that the risk of bleeding is doubled with a PAI–anticoagulant combination compared with monotherapy [44], without any decrease in cardiovascular event incidence [45]. This would also explain why PAI were less prescribed in patients with a history of bleeding in this study.

### 4.3. Limitations

There are some limitations to this study. First it was carried out in a university hospital geriatrics department which is accustomed to managing very frail patients. The monocentric design limits the generalizability of this study seeing that these patients may be managed quite differently in other health care settings and in ambulatory care. Second, the CGA was performed in acute geriatrics during hospitalization for an acute event and may therefore not reflect the patient’s aptitudes before hospitalization. Third, the relatively small sample size is responsible for a potential lack of power and weak associations between PAI prescription and the variables of interest cannot be excluded. Last, frailty was not evaluated by a validated instrument. However, our series has enabled us to provide a comprehensive evaluation of geriatric factors potentially associated with PAI prescription.

## 5. Conclusions

A third of our hospitalized older population had a prescription for PAI on admission. Even though it has proven effective for the prevention of cardiovascular disease, the prescription of PAI was consistent with the patient’s medical history in only 60% of cases. Among patients with an indication for PAI, anticoagulant use and bleeding history were associated with less frequent prescription of PAI. However, age, multimorbidity, motor disabilities, cognitive disorders and malnutrition did not significantly influence prescribing in this geriatric setting. Further studies are needed to confirm these findings.

## Figures and Tables

**Table 1:** Characteristics of patients with an indication for platelet aggregation inhibitors (PAI) (n (%) or mean ± standard deviation).

			No PAI (*n* = 100)	PAI (*n* = 87)	*p*
**Socio-Demographic Characteristics**					
Gender		Female	58 (58)	45 (51.7)	0.438
Age (years)		Mean ± SD	87.4 ± 5.4	87.3 ± 4.9	0.881
		≤80	8 (8)	5 (5.7)	0.774
		81–89	55 (55)	55 (63.2)	0.282
		≥90	37 (37)	27 (31.0)	0.422
Nursing home			23 (23)	16 (18.4)	0.463
**Comorbidities**					
		Chronic heart failure	36 (36)	31 (35.6)	0.958
		Cognitive disorders	50 (50)	40 (46)	0.583
		Chronic kidney disease	5 (5)	7 (8)	0.261
		Chronic respiratory disease	18 (18)	14 (16.1)	0.730
		Diabetes	31 (31)	26 (29.9)	0.869
		Neoplasia	10 (10)	10 (11.5)	0.742
		Peptic ulcer	9 (9)	4 (4.6)	0.238
Multimorbidity		Charlson Index ≥ 2	78 (78)	68 (78.2)	0.988
**Comprehensive Geriatric Assessment**					
	MMSE score	Mean ± SD	17.6 ± 6.1	17.9 ± 7.9	0.782
		≥21	28 (28)	32 (36.8)	0.355
		10–20	49 (49)	27 (31)	0.027
		<10	11 (11)	17 (19.5)	0.227
	Motor skills	Motor disorders	67 (87)	69 (79.3)	0.210
		Falls	38 (38)	27 (31)	0.346
	Nutrition	Serum albumin, (g/L)	28.2 ± 5.5	29.3 ± 4.1	0.136
		Severe malnutrition *	54 (54)	36 (41.4)	0.156
**Co-Prescriptions**					
		Proton pump inhibitors	37 (37)	45 (51.2)	0.043
		Statins	23 (23)	43 (49.4)	<0.001
		SSRI	15 (15)	16 (18.4)	0.534
	Polypharmacy (>5 treatments)		81 (81)	78 (89.7)	0.105
**Cardiovascular History**					
	Primary Prevention		44 (44)	10 (11.5)	0.001
	Secondary Prevention	Coronary artery disease	28 (28)	41 (47.1)	0.006
		Stroke	31 (31)	36 (41.4)	0.124
		Symptomatic PAD	8 (8)	15 (17.2)	0.051
		Other indications **	13 (13)	7 (8)	0.133
**Bleeding Risk**					
	Anticoagulants		57 (57)	7 (8.0)	<0.001
	History of bleeding		12 (12)	8 (9.2)	0.554
	Anemia		47 (47)	42 (48.3)	0.803
	RBC transfusion within 6 months		5 (5)	6 (6.9)	0.757

MMSE: Mini Mental State Examination; PAD: peripheral artery disease; RBC: reb blood cells; SSRI: selective serotonin reuptake inhibitors. * Severe denutrition: according to the Academy Malnutrition Work Group criteria [25]. ** Other indications: other ischemic accident, endovascular procedure, percutaneous aortic valve implantation.

**Table 2:** Multivariate analysis of factors associated with prescription of platelet aggregation inhibitors.

	Odds Ratio	95% CI	*p*
**Age, Comorbidities and Frailty**			
Age (per year)	1.00	0.91–1.08	0.835
Charlson index (per point)	0.97	0.75–1.26	0.803
MMSE (per point)	0.98	0.91–1.06	0.673
Motor disorders	2.01	0.32–13.75	0.438
Falls	1.56	0.60–4.04	0.364
Albumin (g per L)	1.07	0.96–1.18	0.249
Polypharmacy (≥5 treatments)	4.14	1.12–15.29	0.033
**Cardiovascular History**			
Primary prevention	0.04	0.01–0.20	<0.001
Coronary artery disease	1.10	0.28–4.32	0.897
Stroke	0.76	0.19–3.02	0.693
Symptomatic PAD	5.30	1.07–26.2	0.041
Other indication *	0.90	0.02–0.55	0.009
**Bleeding Risk**			
History of bleeding	0.14	0.03–0.63	0.030
Anticoagulants	0.01	0.004–0.05	<0.001

CI: confidence interval, MMSE: Mini Mental State Examination; PAD: peripheral artery disease. * Other indication: other ischemic accident, endovascular procedure, percutaneous aortic valve implantation.
